# A Substance Abuse, Violence and Unhealthy Alcohol Use Syndemic Predicts Depression: A Longitudinal Study of Black Women Living With HIV in Four US Cities

**DOI:** 10.1002/brb3.71609

**Published:** 2026-07-31

**Authors:** T. Dyer, R. Turpin, D. Boyd, M. Mittal, C. Najib, C. Owusu, L. Watson, M. C. Kempf, D. Konkle‐Parker, P. Tien, G. Wingood, T. Neilands, M. Johnson, S. Weiser, E. Arnold, J. Turan, B. Turan, E. Topper, A. Norcini‐Pala

**Affiliations:** ^1^ School of Public Health, Department of Epidemiology and Biostatistics University of Maryland College Park Maryland USA; ^2^ College of Public Health, Department of Global and Community Health George Mason University Fairfax Virginia USA; ^3^ College of Social Work The Ohio State University Columbus Ohio USA; ^4^ School of Public Health, Department of Family Science University of Maryland College Park Maryland USA; ^5^ Centers For Disease Control and Prevention National HIV Surveillance Branch Atlanta Georgia USA; ^6^ Schools of Nursing, Public Health, and Medicine University of Alabama at Birmingham Birmingham Alabama USA; ^7^ Schools of Nursing, Medicine and Population Health University of Mississippi Medical Center Oxford Mississippi USA; ^8^ Department of Medicine, Department of Veteran Affairs Medical Center University of California, San Francisco and Medical Service San Francisco California USA; ^9^ Department of Sociomedical Sciences Columbia University Mailman School of Public Health New York City New York USA; ^10^ Division of Prevention Science, Department of Medicine University of California San Francisco California USA; ^11^ Division of HIV, ID and Global Medicine, Department of Medicine University of California San Francisco California USA; ^12^ School of Public Health University of Alabama at Birmingham Birmingham Alabama USA; ^13^ Department of Epidemiology, Bloomberg School of Public Health Johns Hopkins University Baltimore Maryland USA; ^14^ Department of Community Health Sciences, School of Public Health State University of New York Downstate Health Sciences University Brooklyn New York USA

## Abstract

**Objective:**

Black women living with HIV (BWLH) have a heightened susceptibility to depression due to factors such as intimate partner violence (IPV), alcohol use, and substance abuse. This study examined the relationship between Substance Abuse, Violence, and Heavy Alcohol use (SAVA) among BWLH to determine whether this relationship creates distinct syndemic classes and whether class membership was associated with depressive symptoms.

**Methods:**

Data for this study were drawn from the WAVE sub‐study, which included women enrolled in the WIHS (*n* = 363) collected between April 2016 and April 2017 in four US cities. Latent class analysis was used to identify subgroups based on cannabis use, alcohol use, substance use, and violence. Regression analyses explored the association between baseline syndemic class membership and depressive symptoms at 6, 12, and 18 months.

**Results:**

Three latent classes were identified: “syndemic,” “non‐syndemic low substance use,” and “non‐syndemic moderate cannabis use.” Women in the syndemic class, characterized by higher levels of substance use and violence, had significantly higher depressive symptoms compared to those in the non‐syndemic classes. Longitudinal regression revealed that membership in the syndemic class was associated with a 54% increased risk of depressive symptoms at 6 months, 34% at 12 months, and 51% at 18 months. Bidirectional analyses indicated that baseline violence was associated with increased risk for substance use, and vice versa.

**Conclusions:**

Findings highlight the high prevalence of SAVA syndemic factors and their association with depressive symptoms among BWLH and the need for integrated and culturally competent care that addresses both mental health and syndemic factors to improve outcomes for this vulnerable population.

## Abstract 2

Black women living with HIV (BWLH) experience intersecting risks from substance abuse, violence, and heavy alcohol use (SAVA). Latent class analysis identified three groups, with those in the Syndemic class showing significantly higher depressive symptoms across 6, 12, and 18 months. Findings underscore the need for integrated, culturally responsive interventions addressing syndemic factors and mental health among BWLH.

## 1 Introduction

In the United States, HIV remains a significant health concern among Black women. According to the Centers for Disease Control and Prevention (2023a), Black women in the United States accounted for 54% of all women living with HIV in 2021 (Centers for Disease Control and Prevention 2023b). Despite advancements in prevention and treatment, Black women continue to be disproportionately affected by the virus. Data show that 72.5% of Black women who are living with HIV (BWLH) were less likely to have recent viral suppression than Hispanic and White women (McFall et al. 2013). These data are consistent with previous literature showing that BWLH are less likely to be virally suppressed, with risk factors such as depression and substance use being obstacles to their HIV care engagement and adherence (Lambert et al. 2018; Nelson et al. 2021). Findings underscore the urgent need for targeted interventions and resources to address the unique challenges faced by BWLH.

When examining depressive symptoms across racial groups, Black women are more likely than individuals from other racial and ethnic backgrounds to experience symptoms of depression (S. K. Dale and Safren 2020), and studies have shown that BWLH are particularly at increased risk, with 24%–64% meeting criteria for depression (Logie et al. 2013; Horberg et al. 2008). This heightened susceptibility to depression is significant in that depression detrimentally impacts HIV outcomes, such as adherence to antiretroviral therapy (ART), compromising immune function in people living with HIV (PLWH), and potentially leading to HIV progression (Cook et al. 2002; Starace et al. 2002; Evans et al. 2002; Carrington 2006). However, the research on the mental health needs of Black women, including BWLH, in the United States has been significantly understudied (S. K. Dale and Safren 2020). Throughout history, Black women have faced challenges with underdiagnosis, misdiagnosis, and undertreatment of mental health conditions, contributing to and worsening health disparities (Centers for Disease Control and Prevention 2017). Addressing the mental health needs of Black women, particularly BWLH, is crucial because they face an increased risk of premature health decline and the highest rate of morbidity and mortality from HIV (Lesko et al. 2015; Abrams et al. 2019; Lipira et al. 2020).

Risk factors such as substance use, intimate partner violence (IPV), and alcohol use contribute significantly to poor mental health and the management of chronic diseases, including HIV. Substance use, IPV, and alcohol use are prevalent in the general adult population (Nelson et al. 2021; Centers for Disease Control and Prevention n.d.‐a) and are attributed to a combined $600 billion in health care costs and lost productivity in the United States (Brown and Smith 2016; Centers for Disease Control and Prevention n.d.‐b).

Marijuana use and illicit drugs are oftentimes used to cope with adversities and trauma exposures as well as social and structural barriers related to HIV (Cross et al. 2015; Aralis et al. 2018). Substance use is common among PLWH and is associated with poor HIV outcomes (Sullivan et al. 2015; Sharpe et al. 2004; Stewart et al. 2024). However, there is very little research on substance use among BWLH. In a recent study on mental health and substance use among BWLH in Washington DC, 16.19% of the sample met criteria for substance use disorder (Shahid and Dale 2024). Another study conducted in South Florida among BWLH documented a substance use disorder of 24% in their study (Nydegger and Claborn 2020). A qualitative study of Black women by Nydegger et al. examined cultural factors, structural factors (i.e., housing and employment), past and present adverse life experiences, and individual factors (i.e., substance use to cope with stress, self‐medicating with Black women using substances to cope regarding these factors), as well as coping with structural factors and recent adverse life events (National Coalition Against Domestic Violence (NCADV) 2020b).

Black women are also at significantly higher risk for experiencing IPV and being murdered by an intimate partner compared to women from other racial and ethnic groups. IPV includes physical, emotional, and sexual violence along with intimidation, threats, and coercive control to maintain power (National Coalition Against Domestic Violence 2020a). More than half of non‐Hispanic Black women (53.6%) have experienced lifetime sexual violence, physical violence, and/or stalking by an intimate partner (Jack et al. 2018). The rates of intimate partner homicide are higher for Black women than for White women (The National Center for Victims of Crime (NCVC) 2017). Psychological IPV is the most common type of IPV experienced by women. According to a report by The National Center for Victims of Crime (NCVC) (2017), the percentage of Black women estimated to have experienced psychological IPV was 53.8% and is higher than psychological IPV experienced by White and Latinx women (Li et al. 2014). Any type of IPV (physical, sexual, or psychological) is associated with HIV acquisition in women (Machtinger et al. 2012; Blakely and Grocher 2020). The prevalence of IPV among women living with HIV is close to 55%, which is nearly one and a half times the national prevalence (National Coalition Against Domestic Violence (NCADV) 2020b).

Heavy alcohol use in women, ranging from at‐risk use (> 7 drinks per week) and binge drinking (≥ 4 standard drinks on one occasion) to alcohol use disorder (AUD) (Markkula et al. 2012), is associated with increased risk for violence and injury (Brown and Smith 2016). Increased alcohol consumption is also related to a higher risk of alcohol‐related cancers. For women, consuming two drinks daily is linked to an additional five cases of cancer per 100 women (U.S. Department of Health and Human Services 2025). Alcohol use is common among PLWH and can result in negative mental health consequences (Markkula et al. 2012; Mattisson et al. 2011). Data collected in a 2019 study among BWLH revealed that 54% of participants acknowledged some level of alcohol consumption (Ransome et al. 2017). Additionally, 24% admitted to engaging in unhealthy alcohol use, while 27% reported instances of heavy episodic drinking (Ransome et al. 2017). Black women with AUD have poorer health compared to their White counterparts. Brown and Smith (2016) showed that there was a significant decrease in health as a result of AUD among Black women, but no difference in health was found among White women with and without AUD.

While it is difficult to know whether there are cause–effect relationships between substance use, IPV, and unhealthy alcohol, some evidence suggests that these psychosocial risk factors occur in a cluster, rather than being merely correlated with one another, creating a syndemic. For example, a longitudinal study of military service members found that new‐onset heavy alcohol use, mental disorders, and violence often co‐occurred, with no clear temporal pathway among the three conditions (Braithwaite et al. 2016). Another longitudinal study among those living with HIV in the Veterans Aging Cohort Study (VACS) found that heavy alcohol use, indicated by an AUDIT‐C score ≥ 4, and depressive symptoms were temporally concordant and that eliminating any one factor was associated with the cessation of at least one of the other two. This suggests that these three conditions constitute a unique *syndemic*, particularly for PLWH (Charlson et al. 2016).

A *syndemic* is defined as the co‐occurrence of two or more conditions that interact synergistically to increase the burden of disease outcomes (Turpin et al. 2020). Further, given that each of these conditions, substance abuse, IPV, and unhealthy alcohol use, is associated with poor mental health outcomes, the clustering of these conditions, particularly if the prevalence of clustering is high, may result in a substantial excess risk of experiences of depressive symptoms among women living with HIV (Centers for Disease Control and Prevention; Substance Abuse and Mental Health Services Administration 2022). Black women who experience high levels of trauma, including IPV and other forms of violence, have greater needs for substance use and mental health treatment (Hicks et al. 2007).

Additionally, extant studies show that violence is often accompanied by substance use (Hatcher et al. 2022) with a particularly high prevalence among women. The pharmacological effects of alcohol and certain drugs, like cocaine, can increase the risk of violence. For people with underlying mental illness, these effects can exacerbate psychiatric symptoms, which also increases the risk of violence (McCall et al. 2023). The literature highlights the intersecting, co‐occurring, and mutually reinforcing factors of substance use and violence as determinants of HIV risk (Watson et al. 2024), yet the majority examine HIV outcomes, such as adherence to PrEP or ART, with little attention paid to the reciprocal nature of these two components of Substance Abuse, Violence, and Heavy Alcohol use (SAVA^1^). Theoretically, the relationship between substance use and violence may also be bidirectional, reciprocal, and reinforcing of one another, which is yet to be studied in this population of women living with HIV.

Syndemics are synergistic epidemics that increase the risk for negative health outcomes (Dyer et al. 2020; Turpin et al. 2023; Zilberman and Blume 2005). While the health effects associated with each of these individual conditions are well studied and salient for population health (Baggett et al. 2015; Lundin et al. 2016; Eaton et al. 2013; Markkula et al. 2012; Mattisson et al. 2011; SAMHSA 2015), the health effects of psychosocial syndemics (e.g., substance use, including alcohol and opioid use, and IPV) are understudied and thus not well understood in this priority population of Black women (Nydegger et al. 2021; D'Souza et al. 2021). Further, it is unclear whether such syndemic manifestation differentially affects specific populations, such as BWLH, resulting in elevated depression symptoms, compared to women who endorse individual SAVA components.

Utilizing secondary data from a sub‐study of the Women's Interagency HIV Study (WIHS) called the Women's Adherence and Visit Engagement (WAVE), conducted between April 2016 and April 2017, we explored whether these co‐occurring conditions of Substance Abuse, Violence and Unhealthy Alcohol use (SAVA) create distinct syndemic classes, using latent variable modeling. In the literature, a SAVA syndemic typically refers to Substance Abuse, Violence, and AIDS (Singer et al. 2017); however, since the populations of BWLH are already living with HIV, we operationalized SAVA as Substance Abuse, Violence, and Heavy Alcohol use among this priority population of women, for which this syndemic has yet to be explored.

We hypothesized that each factor, measured at baseline, would co‐occur, creating a SAVA syndemic, and that membership in a high syndemic class would be longitudinally associated with increased levels of depression symptoms, compared to membership in low or no syndemic class. Further, the purpose was to examine cross‐sectionally whether the relationship between substance abuse and IPV was reciprocal, bidirectional, and reinforcing of one another for BWLH. Results will support a holistic, syndemic approach to mitigating factors within the syndemic class, as well as substance use and IPV, to reduce mental health sequelae for BWLH.

## 2 Methods

### 2.1 Participants and Procedures

We utilized data from the WAVE study, which is a sub‐study nested within the Women's Interagency HIV Study (WIHS). WIHS is the oldest cohort study of women living with HIV in the United States (Crockett et al. 2020). In the WAVE sub‐study, data were collected on psychosocial aspects of living with HIV (Norcini Pala et al. 2022; Yousuf et al. 2020). The data were collected annually from women enrolled at four of the following WIHS sites: San Francisco, CA; Atlanta, GA; Birmingham, AL; and Jackson, MS. The current study uses data from the first round of WAVE questionnaires (April 2016–April 2017; *N* = 453). Participants completed an interviewer‐assisted data collection procedure during a separate research visit, with similar methods to those used in the WIHS parent study data collection. For consistency, the data collected through WAVE were linked with data collected through WIHS, which include measures of substance use and data on exposure to violence. While the initial sample consisted of 453 women living with HIV, we included *only* those women who self‐reported and identified as being Black at baseline, who had all four study visits (enrollment, 6, 12, and 18 months), and who were not missing data on the SAVA components, after imputation, resulting in a final analytic sample of *n* = 363 BWLH. All participants provided written informed consent. All study activities were approved by each site's Institutional Review Board.

### 2.2 Measures

#### 2.2.1 Syndemic Variables (SAVA)

Exposures included substance abuse, IPV, and heavy alcohol use as components of a SAVA syndemic.

#### 2.2.2 Substance Abuse

We retained two separate substance abuse measures, which included any cannabis use and use of any hard substances (e.g., cocaine, heroin, methamphetamine, etc.) within the 6 months prior to enrollment. Substance use was measured only at baseline. Therefore, any analyses inclusive of substance use were at baseline only (e.g., SAVA and bidirectionality of substance abuse and IPV).

#### 2.2.3 IPV

IPV was measured as any experienced threat of physical IPV, emotional IPV, and coercive control within the past 6 months. The questionnaire asked women if they had ever been prevented from engaging in specific activities by their partner. For example, “Partner prevented participant from making phone calls”, “Partner prevented participant from continuing education,” “Partner prevented participant from seeing friends”, and “Partner prevented participant from leaving/entering house.” There were two additional measures asking women if “Partner threatened when participant talked about condoms” and “Partner threatened to hurt/kill the participant.” The IPV questions were binary, coded as yes/no. Due to low frequency of responses, these measures were subsequently collapsed to create a measure of “any IPV.” For creating the latent classes, the individual items were used. For the bivariate analyses, the binary variable was used.

#### 2.2.4 Heavy Alcohol Use

The CDC defines unhealthy alcohol use in women as having seven or more drinks in a week (National Center for Chronic Disease Prevention and Health Promotion (NCCDPHP) n.d.; D'Souza et al. 2021). For the measure of alcohol use, the questionnaire asked participants to respond to a question asking how many drinks they had in a week: 0 = *none*, 1 = *less than seven drinks/week*, 2 = *7–14 drinks/week*, 3 = *more than 14 drinks/week*. We dichotomized this measure at the cutoff defined by the CDC such that respondents who endorsed categories 2 or 3 were in the unhealthy alcohol use category, while those who responded 0 or 1 were categorized as having no unhealthy alcohol use.

#### 2.2.5 Outcome Measure Depressive Symptoms

Our outcome was depressive symptoms, measured using the 20‐item Center for Epidemiologic Studies‐Depression Scale (CESD‐20). This is a 20‐item scale that covers several dimensions of depression, including feelings of sadness, undue effort accomplishing tasks, and lack of usual enjoyment. Responses were coded based on the number of days experiencing each item in the past week. Response options range from 0 to 3 for each item (0 = *rarely or none of the time*; 1 = *some or little of the time*; 2 = *moderately or much of the time*; 3 = *most or almost all the time*). Scores range from 0 to 60, with high scores indicating greater depressive symptoms. The CESD‐20 has high internal consistency reliability, evidenced in previous studies utilizing this same subsample of women from the WIHS study (*α* = 0.90) (Norcini Pala et al. 2022). We dichotomized the CESD‐20, such that a score of 16 or greater indicated higher depressive symptomatology and potential for a clinical diagnosis of depression compared to a score of < 16 (i.e., the standard clinical cutoff for depression using this measure) (Zou 2004).

Covariates included categorized age (28–39, 40–49, 50–59, and 60–75), highest education level (less than high school, high school, some college or 2‐year degree, and 4‐year degree or more), and annual household income ($6,000 or less, $6001–$12,000, $12,001–$24,000, $24,001–$36,000, and $36,001 or more). Covariates were selected based on previous WAVE studies (Norcini Pala et al. 2022) as well as associations with violence, substance use, and depression from other extant studies reflected in the literature (Substance Abuse and Mental Health Services Administration 2021; Choi et al. 2015).

### 2.3 Missing Data

Missingness for all items was low (< 10%), with most items having < 5% missingness. This includes participants who did not complete all interview timepoints. To handle missing data, we employed full information maximum likelihood imputation, which was supported by the low level of missingness. Post‐imputation, we retained all observations (*n* = 363).

### 2.4 Latent Class Analysis (LCA)

**FIGURE 1:**
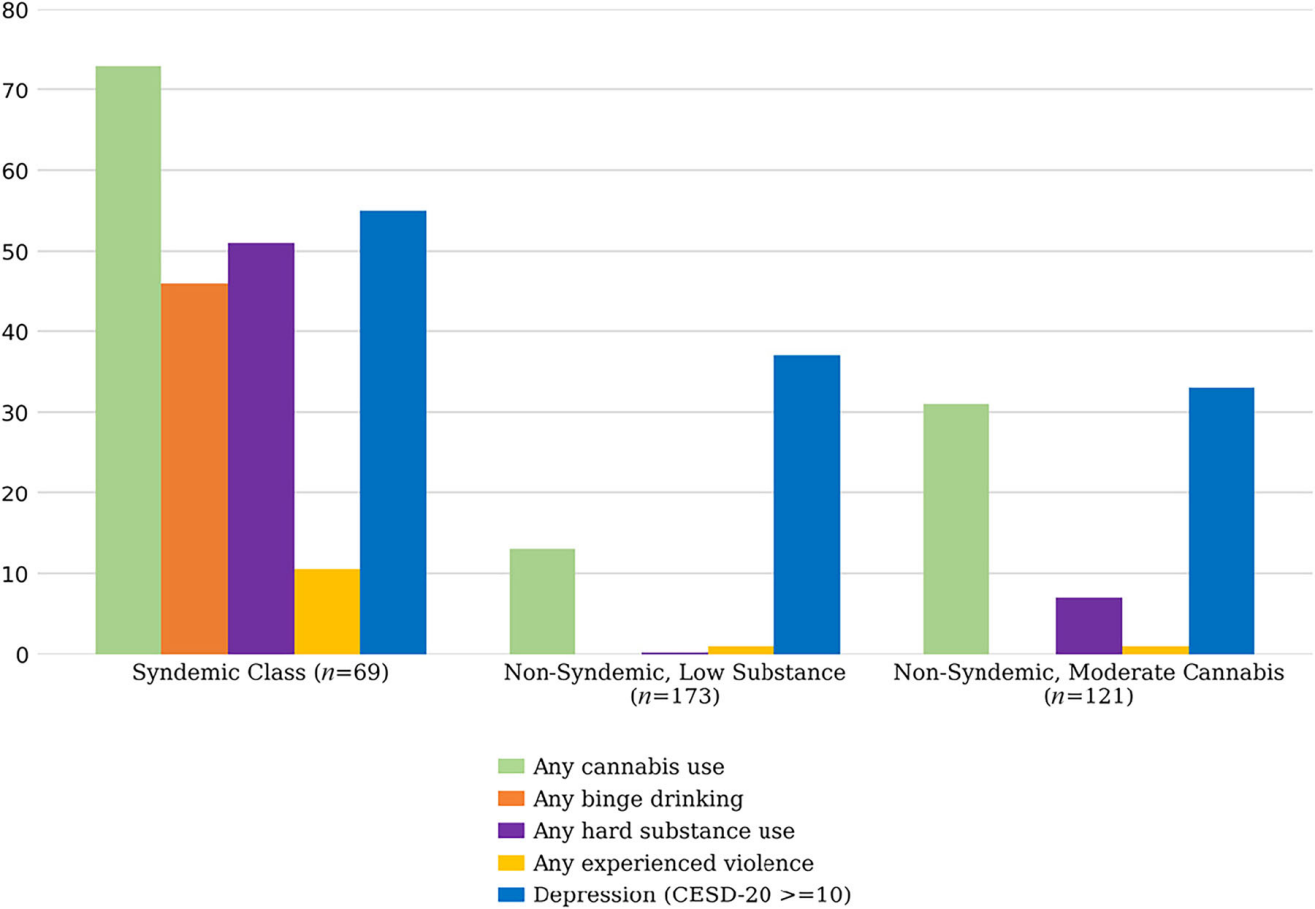
Percentages for syndemic indicators and depression outcome across latent classes (*n* = 363).

We utilized LCA to derive class based on substance use, IPV, and heavy alcohol use indicators, enabling the synthesis of various exposure factors into meaningful person‐centered groups. We did not include depressive symptoms when generating latent class profiles; however, they are presented in Figure 1 to illustrate the outcome visually across classes. The determination of the number of classes was based on several fit criteria, including the log‐likelihood, significant differences between nested models using the Vu‐Mendel–Rubin likelihood ratio test, and information criteria like the Bayesian information criterion (BIC). Additionally, we evaluated entropy for class assignment certainty, with 0.8 or greater indicating sufficient certainty of class assignment, and identified “outlier class” with fewer than 10 participants. Analyses incorporated a probit‐transformed residual to reduce violations of local interdependence. We conducted LCA using the poLCA package in R Statistical Software version 4.1.2 (Volavka and Swanson 2010).

### 2.5 Bivariate Analyses

We tested for associations between latent classes and depression ≥ 16 (a standard depression cutoff for the CESD‐20), as well as all covariates. Kruskal–Wallis tests were used for ordinal and continuous covariates, while chi‐square and Fisher exact tests were used for binary covariates and our dichotomized depression outcome. Fisher exact tests were used where covariates had small frequencies (i.e., cell sizes < 10) necessitating this method. We also present distributions of syndemic indicators and our outcome (depression) across latent classes.

### 2.6 Regression Analyses

Modified Poisson regression with robust standard errors was employed to assess associations between latent classes and our depression outcome (Nwangwu‐Ike et al. 2018). This method is useful for generating prevalence ratios for binary outcomes and allows for more inclusion of confounders than log‐binomial modeling. We generated unadjusted models and models adjusted for age, education, and household income. We also controlled for baseline depression in all regression analyses. Ordinal terms were used for each covariate to maximize model convergence. Associations between baseline latent classes and outcomes at 6, 12, and 18 months are presented. For all models, we generated ratio estimates and 95% confidence intervals.

### 2.7 Bidirectional Analyses

We assessed bidirectional relationships between any experienced violence and any substance use. We tested for associations between baseline substance use and endpoint violence among those with no baseline violence (i.e., controlling for baseline violence) and associations between baseline violence and endpoint substance use among those with no baseline substance use (i.e., controlling for baseline substance use). We generated unadjusted models and models adjusted for age, education, and household income.

### 2.8 Quality Assurance

To detect influential outliers, Cook's distances and leverages were analyzed, revealing no significant outliers. Additionally, there was no evidence of intercollinearity, as indicated by variance inflation factors of < 5 for all models. All bivariate and regression analyses were conducted in SAS 9.4 (Wolford‐Clevenger and Cropsey 2020).

## 3 Results

### 3.1 Sample Characteristics

The subsample of Black women in the WAVE study (*N* = 363) had a median age category of 50–59 years (roughly 40%) and had less than a high school education, with a household income of $6001–$12,000 per annum. Note that 30% of the sample reported cannabis use in the past 6 months, whereas only 9% and 12% reported any heavy alcohol use and any hard substance use, respectively. Approximately 3% reported experiencing any IPV and roughly 40% reported depressive symptoms.

### 3.2 Latent Class Characteristics

All latent class models demonstrated high entropy (Table 1). Because there was no improvement in entropy from a 3‐ to a 4‐class model and no statistically significant improvement in fit from a 3‐ to a 4‐class model, we used a 3‐class model for all analyses. Using our three classes, we created a single 3‐class variable for all subsequent analyses. We observed differences in every syndemic factor across syndemic classes (Figure 1). Class 1 was characterized by the highest number of syndemic factors, including cannabis use, any unhealthy alcohol use, any hard substance use, and any experienced violence. Class 2 was characterized as having low cannabis use, unhealthy alcohol use, hard substance use, and experienced violence factors. Class 3 reflects moderate cannabis use and little to no unhealthy alcohol use or experienced violence. Based on the relative frequency of syndemic factors, we named the classes “syndemic” (*n* = 69), “non‐syndemic low substance use” (*n* = 173), and “non‐syndemic moderate cannabis use (*n* = 121).” Overall, the highest levels of depressive symptoms were observed among the women in the syndemic class, followed by the non‐syndemic moderate cannabis use class, who had the lowest levels of depression.

**TABLE 1:** Fit indices across latent class models with varying numbers of class (*N* = 363).

Class	AIC	BIC	Entropy	Log likelihood	Class *n* < 30
1	4377.253	4455.798	1.000	−2173.627	No
2	3872.178	4034.504	0.945	**−1905.089**	No
3	3727.731	3973.839	0.842	**−1816.866**	No
4	3733.508	4063.397	0.822	−1803.754	No
5	3741.039	4154.71	0.854	−1791.519	Yes

*Note*: Bolded values indicated statistically significant (*p* < 0.05) difference between the model and the model with 1 fewer class using an adjusted Vu‐Mendel–Rubin likelihood ratio test.

Table 2 reports the proportions of syndemic indicators, depression symptoms, and sociodemographic across latent classes. Membership in the syndemic class was associated with significantly lower education and lower income, compared to both the non‐syndemic low substance use and the non‐syndemic moderate cannabis use classes. Regarding the syndemic class indicators, BWLH in the syndemic class had a significantly higher prevalence of cannabis use, hard substance use, unhealthy alcohol use, and any experienced violence compared to BWLH in both the non‐syndemic low substance use and the non‐syndemic moderate cannabis use class. In addition, BWLH in the syndemic class had a significantly greater prevalence of depression symptoms compared to BWLH in both the non‐syndemic low substance use and the non‐syndemic moderate cannabis use classes.

**TABLE 2:** Proportions of syndemic indicators, depression outcomes, and sociodemographics across latent classes (*N* = 363).

	Total (*n* = 363)	Syndemic class (*n* = 69)	Non‐syndemic, low substance (*n* = 173)	Non‐syndemic, moderate cannabis (*n* = 121)
**Age group (%)** ^a^				
28–39	17.6	13.97	14.88	22.72
40–49	29.7	26.2	28.84	32.23
50–59	39.7	45.85	42.79	33.01
60–75	13.0	13.97	13.49	12.04
				
**Highest education level (%)** ^a^				
Less than high school	32.3	**39.3**	**35.97**	**24.66**
High school	30.7	**30.57**	**30.39**	**31.07**
Some college or 2‐year degree	31.0	**24.02**	**29.15**	**36.31**
4‐Year degree or more	6.1	**6.11**	**4.5**	**7.96**
				
**Annual household income (%)** ^a^				
$6000 or less	17.7	**25.76**	**15.5**	**16.89**
$6001–$12,000	39.7	**44.1**	**40.47**	**36.89**
$12,001–$24,000	25.1	**20.09**	**27.13**	**24.85**
$24,001–$36,000	9.5	**3.93**	**9.3**	**12.23**
$36,001 or more	7.9	**6.11**	**7.6**	**9.13**
				
**Any cannabis use (%)** ^b^	30.5	**72.93**	**13.33**	**30.87**
**Any binge drinking (%)** ^b^	8.8	**46.29**	**0.00**	**0.00**
**Any hard substance use (%)** ^c^	12.1	**51.09**	**0.31**	**6.80**
**Any experienced violence (%)** ^c^	2.8	**10.48**	**0.93**	**1.17**
**Depression ≥ 16 (%)** ^b^	**38.84**	**64.63**	**46.2**	**45.24**

^a^ Associations tested using Kruskal–Wallis test. Bolded estimates have *p*‐values < 0.05.

^b^ Associations tested using chi‐square test. Bolded estimates have *p*‐values < 0.05.

^c^ Associations tested using Fisher exact test. Bolded estimates have *p*‐values < 0.05.

### 3.3 Regression Analyses

Table 3 illustrates the results of the regression analysis testing the longitudinal association between baseline syndemic class membership and depressive symptoms at 6, 12, and 18 months. Women in the syndemic class had a 54% increased risk of depressive symptomatology compared to women in the non‐syndemic moderate cannabis class at 6 months. This trend remained constant at both 12‐ and 18‐month follow up. At 12 months, women in the syndemic class had a 34% increased risk of depressive symptoms compared to women in the non‐syndemic moderate cannabis use class and at 18 months had a 51% increase in risk for depressive symptoms, compared to women in the non‐syndemic moderate cannabis use class. There were no significant differences in depressive symptomatology when comparing women in the no‐syndemic low substance use class to women in the no‐syndemic moderate cannabis use class.

**TABLE 3:** Associations between baseline syndemic classes and 6‐, 12‐, and 18‐month depression outcomes (*N* = 363).

	Unadjusted	Adjusted
**6‐month outcomes**		
Syndemic class	**1.62 (1.45, 1.82)**	**1.54 (1.36, 1.75)**
Non‐syndemic low substance	1.15 (0.97, 1.38)	1.15 (0.93, 1.42)
Non‐syndemic moderate cannabis	Reference	Reference
Age group (ordinal)		1.03 (0.92, 1.14)
Highest education (ordinal)		0.92 (0.82, 1.03)
Household income (ordinal)		**0.89 (0.81, 0.98)**
		
**12‐month outcomes**		
Syndemic class	**1.56 (1.30, 1.86)**	**1.34 (1.05, 1.71)**
Non‐syndemic low substance	0.96 (0.70, 1.32)	0.93 (0.67, 1.29)
Non‐syndemic moderate cannabis	Reference	Reference
Age group (ordinal)		1.09 (0.98, 1.22)
Highest education (ordinal)		1.00 (0.88, 1.14)
Household income (ordinal)		**0.77 (0.67, 0.88)**
		
**18‐month outcomes**		
Syndemic class	**1.57 (1.24, 2.00)**	**1.51 (1.23, 1.85)**
Non‐syndemic low substance	1.07 (0.72, 1.57)	1.04 (0.68, 1.57)
Non‐syndemic moderate cannabis	Reference	Reference
Age group (ordinal)		1.07 (0.89, 1.29)
Highest education (ordinal)		0.95 (0.82, 1.11)
Household income (ordinal)		0.97 (0.92, 1.02)

*Note*: Estimates adjusted for age, education level, and household income. Bolded estimates have *p*‐values < 0.05. Underlined estimates have *p*‐values < 0.10 and > 0.05.

Table 4 shows the bidirectional and reciprocating relationship between baseline violence and substance use at 6 months, controlling for baseline substance use. In addition to examining the bidirectionality, we tested the association between baseline substance use and any violence at 6‐month follow‐up, controlling for baseline violence. The results of this analysis revealed a significant association. Controlling for baseline violence, there was a threefold increase in risk for violence at 6 months for those women who also used substances at baseline. The association between baseline violence and substance use at 6 months also revealed a significant association. Controlling for baseline substance use, there was an almost 200% increase in risk for substance use at 6 months for those women who had also reported baseline violence.

**TABLE 4:** Bidirectional associations between any experienced violence and any substance use (*N* = 363).

	Baseline violence controlling for baseline substance use			Baseline substance use controlling for baseline violence	
	Unadjusted	Adjusted		Unadjusted	Adjusted
**Endpoint substance use**	2.95 (0.90, 9.67)	**3.08 (1.01, 9.42)**	**Endpoint Violence**	**3.79 (1.39, 10.31)**	**2.96 (1.16, 7.54)**
**Age group (ordinal)**		1.02 (0.48, 2.17)			0.72 (0.33, 1.59)
**Highest education (ordinal)**		1.09 (0.66, 1.81)			**0.54 (0.42, 0.71)**
**Household income (ordinal)**		0.86 (0.59, 1.25)			**0.58 (0.46, 0.73)**

*Note*: Estimates adjusted for age, education level, and household income. Bolded estimates have *p*‐values < 0.05. Underlined estimates have *p*‐values < 0.10 and > 0.05.

## 4 Discussion

This study highlights the vulnerability of BWLH to depression, particularly among those who experience a syndemic of substance use, violence, and unhealthy alcohol use (SAVA). BWLH face a complex set of challenges that heighten their vulnerability to experiencing symptoms of depression and significantly impact their quality of life. In our study, nearly 40% of the sample reported symptom levels consistent with clinical depression, which is considerably higher than the rate of depression reported in the US general population and among Black women, which is 18.4% and 27%, respectively (National Center for Chronic Disease Prevention and Health Promotion (NCCDPHP)n.d.). Our study results strengthen existing research on depression among BWLH and corroborate the results from other studies on depression among BWLH documenting a higher prevalence of depression among BWLH as well.

The study results also supported our hypotheses and showed a clear class delineation with BWLH who were members of the syndemic class, compared to BWLH in both of the non‐syndemic classes reporting a greater prevalence of depressive symptoms (S. Dale et al. 2014). The results not only showed a significant relationship between the SAVA syndemic factors (e.g., IPV, alcohol use, and substance use) and depression among BWLH, but the trend also held up over time. More specifically, the results indicate that BWLH who fall into the SAVA syndemic class were 54% more likely to exhibit risk factors for clinical depression at 6 months, 34% more likely at 12 months, and 51% increased risk for clinical depression at 18 months. These results align with prior research that highlights the association between IPV, alcohol use, substance use, and depression. Nearly 55% and 27% of women living with HIV experience lifetime and past‐year IPV, respectively (Machtinger et al. 2012; Illangasekare et al. 2012; Hien and Ruglass 2009). Black women report experiencing IPV at significant higher rates than White women (Illangasekare et al. 2013). Studies have established a strong relationship between IPV and poor mental health in the general population and among women living with HIV, including BWLH (Hien and Ruglass 2009; Hutton et al. 2020; SAS Institute Inc. 2023). Heavy alcohol use has been associated with risk for the onset of depressive symptoms and disorders. In one review, regular or heavy drinking in adolescents was shown to be associated with the risk for developing depressive symptoms and disorders (Andresen et al. 1994). In studies of adults, DSM‐IV AUD was associated with risk for the onset of major depressive disorder and with dysthymia (Volavka and Swanson 2010). There is a higher prevalence of depression in individuals living with HIV than in the general population, negatively impacting the quality of life of PLWH (Nanni et al. 2015).

Because the present study was a secondary data analysis, information about specific activities, clinical referrals, or mental health services offered to participants with elevated depressive symptoms was not available in the analytic dataset. Accordingly, we could not evaluate whether depression screening through the parent WAVE/WIHS study was followed by referrals or service engagement. Future studies should capture these screening‐to‐referral processes to clarify how identification of depressive symptoms can be linked to culturally responsive mental health care for BWLH.

The present study is subject to several limitations. First, the WAVE study only enrolled women living with HIV; therefore, we were unable to establish a comparison group of women not living with HIV. As a result, we could not examine whether there are differences in the association between SAVA and depressive symptoms between the two groups. Several studies show that women with HIV report more substance use, violence, and unhealthy alcohol use compared to those not living with HIV, and the manifestation of mental health challenges is also more pronounced. SAVA increases poor health outcomes, mental health issues, and risk‐taking behavior specifically in women living with HIV. Second, substance use was only measured at baseline, so we did not have the ability to examine SAVA production and sustainment over time, along with depression symptoms. Third, the data used for this study were all self‐report, lending itself to information bias, namely, recall bias and social desirability bias.

Despite these limitations, one of the study's most prominent strengths is the longitudinal structure of the data, which allowed for the assessment of risk for depressive symptoms across multiple timepoints, establishing temporality. Another strength of the study is the ability to examine the co‐occurrence of substance use, violence, and unhealthy alcohol use on depressive symptoms.

## 5 Conclusions

Despite the high prevalence of SAVA syndemic factors and their association with depressive symptoms among BWLH, there is a significant lack of services that are responsive to the syndemic and mental health needs of BWLH. It is critical for healthcare providers, counselors, and other practitioners to take a holistic approach when caring for BWLH with depressive symptoms. This includes addressing the social determinants of health, such as systemic racism, poverty, IPV, substance use, and stigma, that may exacerbate poor mental health conditions. Providing culturally competent care and support services that are tailored to the specific needs of Black women can help improve their mental health outcomes and quality of life. Furthermore, creating safe spaces for Black women to share their experiences, seek support, and access resources can be beneficial in helping them cope with the challenges they face. By addressing the unique intersection of HIV and depression in Black women, healthcare providers and support systems can work toward improving their overall health and well‐being.

## Author Contributions

**T. Dyer** served as first author and contributed to writing the original draft, review and editing, visualization, and project administration. R. Turpin conducted the analyses and contributed to writing, review, and editing. **D. Boyd**, M. Mittal, **C. Najib**, **C. Owusu**, and **L. Watson** contributed to writing, review, and editing. M. C. Kempf, **D. Konkle‐Parker**, **P. Tien**, **G. Wingood**, **T. Neilands**, **M. Johnson**, **S. Weiser**, **E. Arnold**, **J. Turan**, **B. Turan**, and **E. Topper** provided review and feedback. **A. Norcini‐Pala** served as senior author and contributed to writing, review, and editing.

## Funding

The funding for this work comes from the following funding sources: Typhanye V. Dyer (National Institute on Mental Health, R25MH067127; Centers for Disease Control and Prevention, Prevention Research Centers, U48DP006382; National Heart Lung and Blood Institute, R01HL165686; National Institute on Mental Health, R15MH129191; Gilead Sciences, CO‐US‐412‐6417; National Institute on Minority Health; and Health Disparities Loan Repayment Program, 1L60MD019864), Rodman E. Turpin (National Institute on Minority Health and Health Disparities, K01MD016346), Donte T. Boyd (National Institute of Mental Health, 1R21MH134643‐01), and Andrea Norcini Pala (K01MH125724 and R01MH131177 National Institutes of Health, and the National Institute of Mental Health).

## Ethics Statement

This study was reviewed by the University of Maryland College Park Institutional Review Board and was determined not to meet the definition of human subjects research under federal regulations (Project ID: [2301044‐1]; Decision Date: March 5, 2025). Therefore, IRB approval and informed consent were not required.

## Conflicts of Interest

The authors declare no conflicts of interest.

## Data Availability

Some data from the MACS/WIHS Combined Cohort Study are publicly available through the publicly available dataset that can be requested on the MWCCS webpage. Additional study data used for this analysis are subject to access restrictions and may be obtained by submitting a concept sheet and receiving approval through the MACS/WIHS Combined Cohort Study data request process.

## Endnotes

1 The SAVA acronym used in this paper refers specifically to the co‐occurrence of Substance Abuse, Violence, and Heavy Alcohol use, and is distinct from Singer's model of SAVA.
